# Nucleoside Diphosphate Kinase B Contributes to Arrhythmogenesis in Human-Induced Pluripotent Stem Cell-Derived Cardiomyocytes from a Patient with Arrhythmogenic Right Ventricular Cardiomyopathy

**DOI:** 10.3390/jcm9020486

**Published:** 2020-02-10

**Authors:** Fanis Buljubasic, Ibrahim El-Battrawy, Huan Lan, Santosh K. Lomada, Anupriya Chatterjee, Zhihan Zhao, Xin Li, Rujia Zhong, Qiang Xu, Mengying Huang, Zhenxing Liao, Siegfried Lang, Lukas Cyganek, Xiaobo Zhou, Thomas Wieland, Martin Borggrefe, Ibrahim Akin

**Affiliations:** 1 First Department of Medicine, Medical Faculty Mannheim, University Medical Centre Mannheim, University of Heidelberg, Theodor-Kutzer-Ufer 1-3, 68167 Mannheim, Germany; fanis.buljubasic@umm.de (F.B.); Ibrahim.el-battrawy@medma.uni-heidelberg.de (I.E.-B.); Zhihan.Zhao@medma.uni-heidelberg.de (Z.Z.); Xin.Li@medma.uni-heidelberg.de (X.L.); rujia.zhong@medma.uni-heidelberg.de (R.Z.); qiang.xu@medma.uni-heidelberg.de (Q.X.); Mengying.Huang@medma.uni-heidelberg.de (M.H.); Liao.Zhenxing@medma.uni-heidelberg.de (Z.L.); Siegfried.Lang@medma.uni-heidelberg.de (S.L.); martin.borggrefe@umm.de (M.B.); Ibrahim.Akin@umm.de (I.A.); 2 DZHK (German Center for Cardiovascular Research), Partner Sites, Heidelberg-Mannheim and Göttingen, 68167 Mannheim, Germany; lukas.cyganek@gwdg.de (L.C.); Thomas.Wieland@medma.uni-heidelberg.de (T.W.); 3 Key Laboratory of Medical Electrophysiology of Ministry of Education and Medical Electrophysiological Key Laboratory of Sichuan Province, Institute of Cardiovascular Research, Southwest Medical University, 646000 Luzhou, China; lh6402196@126.com; 4 Experimental Pharmacology Mannheim, European Center for Angioscience, Medical Faculty Mannheim, University of Heidelberg, 68167 Mannheim, Germany; Santosh.Lomada@medma.uni-heidelberg.de (S.K.L.); Anupriya.Chatterjee@medma.uni-heidelberg.de (A.C.); 5 Stem Cell Unit, Clinic for Cardiology and Pneumology, University Medical Center Göttingen, 37075 Göttingen, Germany

**Keywords:** arrhythmogenic right ventricular cardiomyopathy, human-induced pluripotent stem cell-derived cardiomyocyte, nucleoside diphosphate kinase, SK4 channel, arrhythmia

## Abstract

Background: Arrhythmogenic right ventricular cardiomyopathy (ARVC) is a rare, inheritable cardiac disorder characterized by ventricular tachyarrhythmias, progressive loss of cardiomyocytes with fibrofatty replacement and sudden cardiac death. The exact underlying mechanisms are unclear. Methods: This study investigated the possible roles of nucleoside diphosphate kinase B (NDPK-B) and SK4 channels in the arrhythmogenesis of ARVC by using human-induced pluripotent stem cell-derived cardiomyocytes (hiPSC-CMs). Results: In hiPSC-CMs from a patient with ARVC, the expression levels of NDPK-B and SK4 channels were upregulated, the cell automaticity was increased and the occurrence rate of arrhythmic events was enhanced. Recombinant NDPK-B applied into hiPSC-CMs from either healthy donors or the patient enhanced SK4 channel current (I_SK4_), cell automaticity and the occurrence of arrhythmic events, whereas protein histidine phosphatase 1 (PHP-1), a counter actor of NDPK-B, prevented the NDPK-B effect. Application of PHP-1 alone or a SK4 channel blocker also reduced cell automaticity and arrhythmic events. Conclusion: This study demonstrated that the elevated NDPK-B expression, via activating SK4 channels, contributes to arrhythmogenesis in ARVC, and hence, NDPK-B may be a potential therapeutic target for treating arrhythmias in patients with ARVC.

## 1. Introduction

Arrhythmogenic right ventricular cardiomyopathy (ARVC) is an inheritable cardiac disorder characterized by ventricular tachyarrhythmias, progressive loss of cardiomyocytes with fibrofatty replacement and sudden cardiac death (SCD) [1]. The prevalence of ARVC is about 1:2000–1:5000 and more common in males (2:1–3:1) [1]. ARVC usually manifests at ages between 12 to 60 years and is a leading cause of SCD due to ventricular tachyarrhythmias in young athletes [2]. In the most typical form of ARVC, the right ventricle is primarily affected. As the disease progresses, the left ventricle may also be affected [3].

Most cases of ARVC are attributed to mutations in desmosomal genes, including plakoglobin (JUP), plakophilin-2 (PKP2), desmoplakin (DSP), desmoglein-2 (DSG2) and desmocollin-2 (DSC2) [4]. However, the exact pathogenic mechanisms by which desmosomal mutations cause life-threatening arrhythmias remain unclear. To date, two hypotheses have been discussed to explain the arrhythmogenic mechanisms in ARVC: (1) structural abnormality-induced conduction-defects; (2) ion channel dysfunction-induced electrophysiological abnormalities. The former is caused mainly by an intercellular fibrofatty deposit, which interrupts the intercellular propagation of electrical pulses. The latter can be caused by ion channel remodeling, leading to a gain-of-function or a loss-of-function of ion channels and subsequent abnormal electrical activity in cardiomyocytes. For example, the peak sodium current (I_Na_) has been shown to be reduced in ARVC-cardiomyocytes [5]. The reduced I_Na_ may slow the propagation of excitation, leading to a functional conduction defect. El-Battrawy et al. showed that human-induced pluripotent stem cell-derived cardiomyocytes (hiPSC-CMs) from a patient with ARVC (ARVC-hiPSC-CMs) carrying a mutation in the DSG2 gene displayed multiple ion channel dysfunctions and abnormal electrical activities [6], pointing to the contribution of ion channel dysfunctions to arrhythmogenesis, independent of structural abnormalities. However, the exact mechanisms by which the conduction defect or ion channel dysfunctions cause tachyarrthmias in patients with ARVC have not been clearly clarified.

Some calcium activated K^+^ channels, including small conductance (SK1, SK2, SK3) and intermediate conductance (SK4, also called KCa3.1) calcium-activated K^+^ channels, have been linked to arrhythmogenesis in atrial fibrillation (AF) and catecholaminergic polymorphic ventricular tachycardia (CPVT) [7,8]. The SK1–3 channel currents can change AP-duration (APD) [7], whereas SK4 channel currents can influence the pacemaker activity of cells [9]. Both may enhance the occurrence of arrhythmias in cardiomyocytes. Whether those ion channels, especially the SK4 channels, are also involved in arrhythmogenesis in ARVC, is not known.

It is well-known that besides intracellular Ca^2+^ ions, nucleoside diphosphate kinase B (NDPK-B) is an important intracellular regulator of SK4 channels [10,11]. NDPK-B can directly phosphorylate SK4 channels at histidine 358 (His358) and enhance channel activity [12]. In general, nucleoside diphosphate kinases (NDPKs) are ubiquitously expressed nucleoside 5′-triphosphate (NTP)/nucleoside 5′-diphosphate (NDP) transphosphorylases. They are encoded by the NME (nonmetastatic cell) genes, which comprise a family of 10 related genes. Among them, the class I subfamily (consisting of NDPK-A, B, C and D) exerts enzymatic activity [13]. NDPK-A and NDPK-B are involved in various cellular processes, including proliferation, differentiation, development and metastasis [14]. In addition, NDPK-B acts as a mammalian protein histidine kinase by transferring the phosphate from its high energy phosphate intermediate to histidine residues on other proteins [11]. Besides His358 in SK4 channels, another well-characterized NDPK-B substrate is His266 in the β-subunit of heterotrimeric G proteins [11,15]. Activation of the G protein by NDPK-B (resulting from phosphorylation of His266) increases intracellular cAMP formation independent of receptor activation. This connects NDPK-B to various physiological and pathophysiological cAMP-related processes in cells such as cardiomyocytes. Indeed NDPK-B has been linked to different diseases, including heart failure [16]. Whether NDPK-B also plays a role in arrhythmogenic mechanisms in patients with ARVC has not been examined so far. Given that SK4 channels are important for pacemaker activity and are activated by NDPK-B, we designed this study to assess a possible involvement of SK4 channels and NDPK-B in the arrhythmogenesis of ARVC.

## 2. Materials and Methods

### 2.1. Ethics Statement

The skin biopsies from two healthy donors and one ARVC patient were obtained with written informed consent. The study was approved by the Ethics Committee of the Medical Faculty Mannheim, University of Heidelberg (approval number: 2018-565N-MA) and by the Ethics Committee of the University Medical Center Göttingen (approval number: 10/9/15). The study was carried out in accordance with the approved guidelines and conducted in accordance with the Helsinki Declaration of 1975 (https://www.wma.net/what-we-do/medical-ethics/declaration-of-helsinki/), revised in 2013.

### 2.2. Generation of Human iPS Cells and iPS Cell-Derived Cardiomyocytes (hiPSC-CMs)

The hiPS cells and hiPSC-CMs were generated from the same healthy donors (Donor 1 and Donor 2) and from the same patient with ARVC as described in earlier study [6]. Briefly, human iPS cells (hiPSCs) were generated from primary human fibroblasts derived from a skin biopsy. The hiPSC line was generated in feeder free culture conditions using the integration-free CytoTune-iPS 2.0 Sendai Reprogramming Kit (Thermo Fisher Scientific, Schwerte, Germany #A16517) with the reprogramming factors OCT4, KLF4, SOX2 and c-MYC according to manufacturer’s instructions, with modifications. To prove the success of hiPS cell generation, the generated hiPSCs were characterized for their pluripotency and their in vitro differentiation potential, which have been shown in our recent study [6].

The generation of hiPSC-CMs has been described in our previous studies. Briefly, culture dishes and wells for hiPSCs were coated with Matrigel (Corning, Kaiserslautern, Germany). The culture medium TeSR-E8 (Stemcell Technologies, Köln, Germany) was used for hiPSCs, and RPMI 1640 Glutamax (Life Technologies, Darmstadt, Germany) containing sodium pyruvate, penicillin/streptomycin, B27 (Life Technologies) and ascorbic acid (Sigma Aldrich, Taufkirchen, Germany) was used for hiPSC-CMs. During the first 3 weeks, CHIR99021 (Miltenyi Biotec, Bergisch Gladbach, Germany ), BMP-4 (R&D Systems, Wiesbaden, Germany), Activin A (R&D Systems), FGF-2 (Miltenyi Biotec) and IWP-4 (Miltenyi Biotec) were added at different time points to induce the cells to differentiate into hiPSC-CMs. During the third week a lactate (Sigma Aldrich) containing RPMI-medium without glucose and glutamine (Biological Industries, Cromwell, IN, USA) was added for selecting cardiomyocytes. At 40 to 60 days of culture with basic culture medium, cardiomyocytes were dissociated from 24 well plates and plated as single cells on matrigel-coated 3.5 cm petri dishes for patch-clamp measurements. The cells from this ARVC patient carried a missense mutation (p.Gly638Arg) in the desmoglein-2 (DSG2) gene.

To prove the successful differentiation of hiPSC-CMs, the expression of different cardiac markers was assessed at mRNA and protein levels, which have been shown in our recent study [6]. The hiPSC-CMs used in this study were generated by the same differentiation protocol as used in the previous study and displayed similar cardiac features (Figure S1).

### 2.3. Polymerase Chain Reaction Assays

The preparation of total RNA using the RNeasy mini kit (Qiagen, Hilden, Germany), including DNAse treatment, was performed by the following protocol. The cDNA was amplified by qPCR on StepOnePlus Real-Time PCR System (Applied Biosystems, Thermo Fisher Scientific, Waltham, MA, USA) using a PCR mix with hot start Taq DNA polymerase and SYBR Green (Sibir Rox Hot Mastermix, BIORON, Römerberg, Germany; Cat number 119405) in the presence of sense and antisense primers (400 nM each, RT^2^ qPCR Primer Assays from Qiagen, Germany). Relative mRNA expression level was calculated as the expression of the mRNA of the gene of interest relative to GAPDH in samples from treated or untreated (control) cells. The expression level was calculated by the ΔΔCT method, based on the threshold cycle (CT), as fold change = 2^−Δ(ΔCT)^, where ΔCT = CT_gene of interest_ − CT_GAPDH_ and Δ(ΔCT) = ΔCT_treated_ − ΔCT_control_. Results are shown as means ± SEMs from the measurements of at least 3 biological replicates and 2 technical replicates.

### 2.4. Western Blot

The cell lysates of hiPSC-CMs were used for protein isolation. Western blotting was performed using proteins extracted with RIPA buffer (50 mM Tris-HCl, pH 7.4, 150 mM NaCl, 1 mM dithiothreitol, 1% Triton X-100, 1% sodium deoxycholate). The proteins were separated by SDS-PAGE and electrically transferred onto nitrocellulose membranes. After blocking with Roti-block (Roth, Karlsruhe, Germany), membranes were incubated with primary antibodies overnight. Immunocomplexes were incubated with corresponding secondary antibodies and visualized using a chemiluminescent peroxidase substrate (Roche, Mannheim, Germany; or Thermo Scientific, Rockford, IL, USA). Protein expression was quantified using Image J (NIH, Bethesda, MD, USA). Specific primary antibodies used were mouse-anti-NDPK-B (MC-412; Kamiya, Seattle, WA, USA), mouse-anti-γ-tubulin (Sigma-Aldrich) and mouse-anti-SK4 (sc-365265, Santa Cruz Biotechnology, Heidelberg, Germany).The corresponding secondary antibody was rabbit anti-mouse peroxidase (Sigma-Aldrich).

### 2.5. Recombinant NDPK Isoforms and PHP-1

Expression of His6-tagged NDPK-A, NDPK-B and NDPK-C as well as His6-tagged PHP-1 in and purification from *Escherichia coli* was described in detail before [11].

After the purification of NDPKs, their enzyme activity was confirmed by measuring the transphosphorylase activity (Figure S2). The transphosphorylase activity of the NDPKs was measured in a reaction mixture containing 50 mM Tris-HCl, pH 7.5, 2 mM MgCl_2_, 1 mM DTT and 0.01% BSA (Buffer A). Stock proteins were diluted in Buffer A to a concentration of 300 pM, and 1 volume of the rNDPK solution was mixed with 1 volume of substrate mixture (200 µM GTP and 20 µM ADP in Buffer A) on a 384 well plate. The mixture was incubated at room temperature for 30 min, and 2 volumes of the Kinase-GLO reagent (Promega, Walldorf, Germany, V6711), containing an ATP dependent firefly luciferase, were added. The luminescence was measured using a plate reader (PerkinElmer-EnVision, Baesweiler, Germany). Six different concentrations of ATP (0–10 µM) were used as standards for a calibration curve to calculate the amount of ATP produced by the different NDPKs. All readings shown were obtained under conditions where the ATP formation was still linear with time and enzyme concentration.

The activity of PHP-1 was proven by functional assessments in our previous study, showing that PHP-1 abolished NDPK-B effects, but the enzyme-inactive mutant PHP-1 failed to do so, indicative of the enzyme activity of PHP-1 [17].

### 2.6. Patch-Clamp Recordings

The whole-cell patch-clamp recordings (voltage and current-clamp configurations) were carried out at room temperature (22–25 °C). To isolate the I_SK4_ TRAM-34 (1 µM) or clotrimazole (3 µM) was added in the bath solution containing 130 mM/L NaCl, 5.9 mM/L KCl, 2.4 mM/L CaCl_2_, 1.2 mM/L MgCl_2_, 11 mM/L glucose and 10 mM/L HEPES (pH 7.4 (NaOH)). The pipette solution contained 10 mM HEPES, 126 mM KCl, 6 mM NaCl, 1.2 mM MgCl_2_, 5 mM EGTA, 11 mM glucose, and 1 mM MgATP (pH 7.4 (KOH)). In addition, appropriate CaCl_2_ was added to get the free Ca^2+^ concentration of 0.5 μM according to the calculation by the software MAXCHELATOR (http://web.stanford.edu/~cpatton/downloads.htm). TRAM-34 sensitive currents were evaluated as I_SK4_. In experiments, to assess the effects of NDPK-B and PHP-1, NDPK-B (30 ng/mL) alone or with PHP-1 (100 ng/mL), or PHP-1 alone, was added into cells through the recording pipette solution. To minimize the effects of rundown of recorded currents on the results of experiments, we carefully monitored the time-dependent change of currents. Recordings were started when the current became stable, usually within 3 to 5 min. In spontaneous action potential (AP) recordings the extracellular and intracellular solutions were the same as used for current measurements.

### 2.7. Drugs

Clotrimazol, ivabradin, mibefradil and nifedipine were purchased from Sigma. TRAM-34 was purchased from Tocris Bioscience.

### 2.8. Statistical Analysis

If not otherwise indicated, data are shown as means ± SEMs and were analyzed using InStat© (GraphPad, San Diego, CA, USA) and SigmaPlot 11.0 (Systat GmbH, Erkrath Germany). By analyzing the data with the Kolmogorov Smirnov test, it was decided whether parametric or non-parametric tests were to be used for analysis. Student’s *t*-test and the Mann–Whitney U-test were used to compare continuous variables with normal and non-normal distributions, respectively. To compare categorical variables, the Fisher-test was used. For parametric data of more than two groups, one-way ANOVA with a Bonferroni post-test for multiple comparisons was performed. For non-parametric data, the Kruskal-Wallis test with Dunn’s multiple comparisons post-test was used. An unpaired Student’s *t*-test was used for comparisons of two independent groups with normal distributions. The paired t-test was used for comparisons of data before and after application of a drug. *p* <0.05 (two-tailed) was considered significant.

## 3. Results

### 3.1. The Expressions of NDPK-B and the SK4 Channel Were Both Increased in ARVC-hiPSC-CMs.

**Figure 1:**
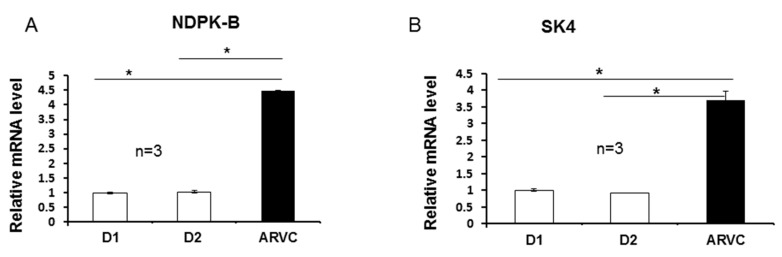

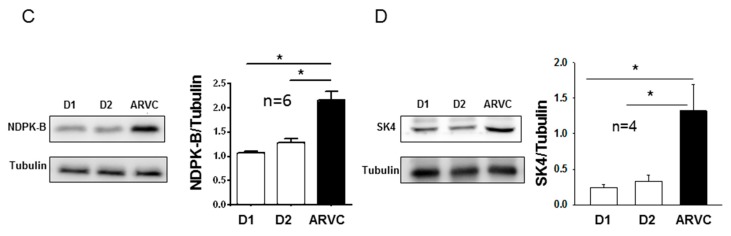
Expressions of nucleoside diphosphate kinase B (NDPK-B) and SK4 channel were increased in arrhythmogenic right ventricular cardiomyopathic (ARVC)-human-induced pluripotent stem cell-derived cardiomyocytes (hiPSC-CMs). Quantitative polymerase chain reaction and Western blot analyses were performed in hiPSC-CMs derived from healthy donors (D1, D2) and the ARVC-patient. (**A**,**B**) Relative mRNA levels of the gene expression (normalized to the housekeeping gene GAPDH) of NDPK-B (**A**) and SK4 channel (**B**). (**C**) Representative examples (left panel) and statistical data (right panel) of Western blots of cell lysates from both donor cell lines (D1, D2) and the ARVC cell line, showing the protein expression levels of NDPK-B. The intensity of the immunoreactive bands of NDPK-B protein (20 kD) was normalized to that of tubulin. (**D**) Representative examples (left panel) and statistical data (right panel) of Western blots of cell lysates from both donor cell lines (D1, D2) and the ARVC cell line, showing the expression levels of the SK4 protein (45 kD). * *p* < 0.05; n represents number of experiments.

Our recent study demonstrated that hiPSC-CMs from the ARVC-patient were more susceptible than donor cells to adrenergic stimulation and showed more arrhythmic events [6]. To determine a possible involvement of NDPK-B and SK4 channels in arrhythmogenesis of ARVC, in this study we first checked the expression levels of NDPK-B and SK4 in hiPSC-CMs from the ARVC patient and from the healthy donors. The results from qPCR and Western blot analysis displayed that both the mRNA and protein levels of NDPK-B and SK4 channels were higher in ARVC-hiPSC-CMs than those in donor-hiPSC-CMs (Figure 1), suggesting possible roles of NDPK-B or SK4 in pathogenesis of ARVC.

### 3.2. SK4 Channel Currents, Pacemaker Activity and the Occurrence of Arrhythmic Events Were Enhanced in ARVC-hiPSC-CMs

To examine the possible consequences of upregulation of NDPK-B and SK4 channels in ARVC-hiPSC-CMs, the SK4 channel currents (I_SK4_), pacemaker activity and arrhythmic events were assessed in hiPSC-CMs.

**Figure 2:**
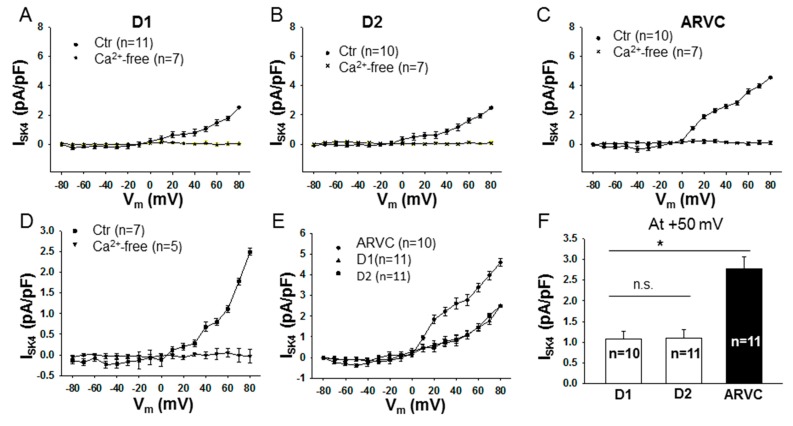

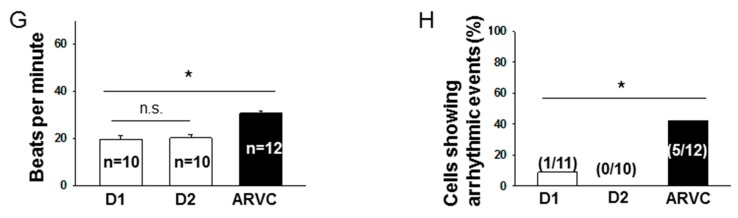
SK4 channel currents, and beating frequency and occurrence rate of arrhythmic events, were higher in ARVC-hiPSC-CMs than in donor cells. SK4 channel currents (I_SK4_) were measured in whole-cell configuration in healthy donor (D1, D2) or ARVC cells depolarized from −80 mV to +80 mV for 400 ms with a holding potential of −40 mV. TRAM-34 (1 µM, **A**–**C**,**E**,**F**) and clotrimazole (3 µM, **D**), SK4 channel blockers, were used to separate I_SK4_ from other ion channel currents. For assessing the pacemaker activity and arrhythmic events (early or delayed afterdepolarizations or triggered beats), spontaneous action potentials (sAPs) were recorded in current-clamp mode. (**A**–**C**) The current-voltage relation (I-V) curves of I_SK4_ (TRAM-34-sensitive currents) in hiPSC-CMs from donor one (D1, **A**), donor two (D2, **B**) and the ARVC-patient (ARVC, **C**) with 500 nM Ca^2+^ (Ctr) or without Ca^2+^ (Ca^2+^-free) in the pipette solution. (**D**) I–V curves of clotrimazole-sensitive currents in donor-hiPSC-CMs (D1 cells). Of note, some I–V curves in A-D contain small inward currents, which changed the reverse potential of curves. (**E**, **F**) Comparison of I_SK4_ between donor and ARVC cells showing increased I_SK4_ in ARVC-hiPSC-CMs. (**G**) Frequency of sAPS in donor and ARVC cells showing enhanced pacemaker activity in ARVC-hiPSC-CMs. (**H**) Percentage of cells showing arrhythmic events in donor and ARVC cells. * *p* < 0.05; n.s., not significant. The numbers given represent cell numbers.

The whole-cell current recordings plus application of SK4 channel blockers TRAM-34 (1 µM, Figure 2A–C, E–F) and clotrimazole (3 µM, Figure 2D) revealed the presence of I_SK4_ in hiPSC-CMs (Figure 2A–D). In cells with Ca^2+^-free intracellular solution, no TRAM-34- or clotrimazole-sensitive currents could be detected (Figure 2A–D, Ca^2+^-free). These data confirmed the functional expression SK4 channels in sarcolemmal membrane of hiPSC-CMs. Of note, I_SK4_ was larger in ARVC-hiPSC-CMs than that in donor cells (Figure 2E, F), suggesting a possible effect of NDPK-B on I_SK4_ because NDPK-B is upregulated in ARVC cells.

Since SK4 channels have been linked to pacemaker activity and arrhythmias [8,9], we compared the pacemaker activity and occurrence rate of arrhythmic events between donor and ARVC-hiPSC-CMs. It was indeed detected that ARVC-hiPSC-CMs possessed higher pacemaker activity (higher frequency of APs) and displayed more arrhythmic events (Figure 2G–H).

### 3.3. Recombinant NDPK-B Activated the SK4 Channels, Enhanced Pacemaker Activity and Increased the Arrhythmic Events in hiPSC-CMs

**Figure 3:**
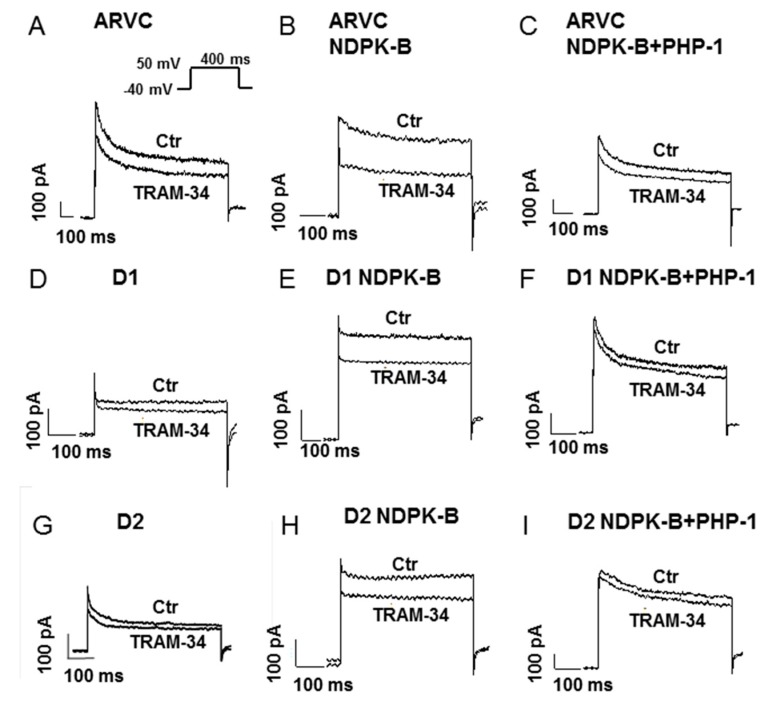
Representative SK4 currents in hiPSC-CMs. SK4 channel currents (I_SK4_) were measured in whole-cell configuration in cells depolarized from −40 mV to +50 mV for 400 ms. TRAM-34 (1 µM) was used to separate I_SK4_ from other ion channel currents. TRAM-34-sensitive currents represent I_SK4_. Recombinant NDPK-B (30 ng/mL), either alone (**B**,**E**,**H**) or with PHP-1 (100 ng/ml, **C**,**F**,**I**), was applied into healthy donor (D1, D2) or ARVC cells though the patch pipette. (**A**–**C**) Representative currents in ARVC-hiPSC-CMs in the absence (Ctr) and presence of TRAM-34. (**D**–**F**) Representative currents in D1-hiPSC-CMs in the absence (Ctr) and presence of TRAM-34. (**G**–**I**) Representative currents in D2-hiPSC-CMs in the absence (Ctr) and presence of TRAM-34.

**Figure 4:**
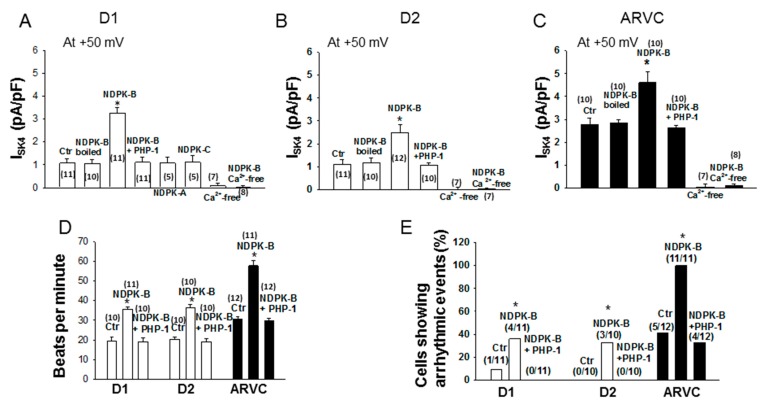
Recombinant NDPK-B enhanced Sk4 channel currents, and beating frequency and occurrence rate of arrhythmic events. Recombinant NDPK (NDPK-B, NDPK-A, NDPK-C, each 30 ng/mL) either alone or with PHP-1 (100 ng/mL) was applied into healthy donor (D1, D2) or ARVC cells though the patch pipette. I_SK4_ or spontaneous action potentials (sAPs) were measured 3 min after whole-configuration was established. The intracellular Ca^2+^ concentration was 500 nM. (**A**–**C**) Mean values of I_SK4_ in donor and ARVC-cells showing that NDPK-B but not the inactivated (boiled) kinase or NDPK-A or NDPK-C enhanced I_SK4_ and PHP-1 abolished the NDPK-B effects. (**D**) Beating frequencies in donor and ARVC cells showing higher frequencies with NDPK-B. (**E**) Occurrence rate of arrhythmic events (early or delayed afterdepolarizations or triggered beats or bigeminy-like or torsade de pointes-like events) in donor and ARVC cells influenced by NDPK-B. * *p* < 0.05 versus control (Ctr). The numbers given in parentheses represent cell numbers.

**Figure 5:**
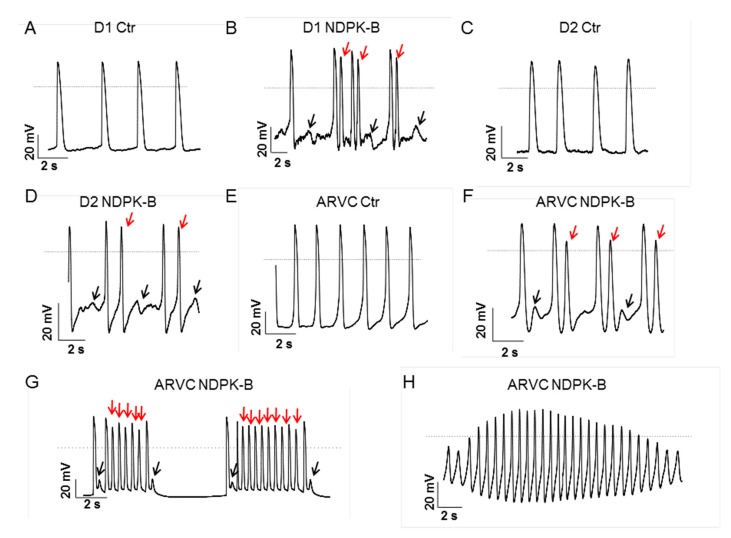
Recombinant NDPK-B induced different forms of arrhythmic events. Recombinant NDPK-B (30 ng/mL) was applied into healthy donor (D1, D2) or ARVC cells though the patch pipette. Spontaneous action potentials (sAPs) were measured in donor and ARVC cells. NDPK-B induced delayed afterdepolarizations (DADs, black arrows) or triggered beats (red arrows). (**A**,**B**) Examples of sAPs in hiPSC-CMs from donor one (D1) without (**A**, Ctr) and with NDPK-B (**B**) showing DADs and bigeminy-like arrhythmic evens. (**C**,**D**) Examples of sAPs in hiPSC-CMs from donor two (D2) without (**C**, Ctr) and with NDPK-B (**D**) showing DADs and bigeminy-like arrhythmic evens. (**E**,**F**) Examples of sAPs in hiPSC-CMs from the ARVC-patient (ARVC) without (**E**, Ctr) and with NDPK-B (**F**) showing DADs and bigeminy-like arrhythmic evens. (**G**) An example of sAPs in ARVC-hiPSC-CMs with NDPK-B showing sustained triggered activity. (**H**) An example of sAPs in ARVC-hiPSC-CMs with NDPK-B showing torsade de pointes-like arrhythmic events.

Since NDPK-B can activate SK4 channels [10,12,17], its effect on I_SK4_ in hiPSC-CMs was checked. Recombinant NDPK-B (30 ng/mL) was applied through the patch pipette into the cells and I_SK4_ was recorded. In donor and ARVC cells, the recombinant NDPK-B enhanced I_SK4_ by 1.5–2 folds (Figure 3A,B,D,E,G,H; Figure 4A–C). Denatured NDPK-B (boiled for 20 min) failed to exert an effect on I_SK4_. The addition of PHP-1 (100 ng/mL), counteracting histidine phosphorylation by NDPK-B, suppressed the enhancing effect of NDPK-B on the I_SK4_ (Figure 4A–C). These results indicate that the NDPK-B-induced activation of I_SK4_ resulted most likely from histidine phosphorylation in SK4 channels. In accordance with previous reports [17], neither NDPK-A nor NDPK-C exerted any effects on SK4 channel currents (Figure 4A). As expected from the Ca^2+^-dependency of SK4 channels, in cells with Ca^2+^-free intracellular solution, NDPK-B did not enhance I_SK4_. In addition, NDPK-B elevated the cell beating frequency (Figure 4D) and the occurrence rate of arrhythmic events (Figure 4E and Figure 5). Application of NDPK-B led to delayed afterdepolarizations or triggered beats or bigeminy-like events in both donor and ARVC-hiPSC-CMs, but the sustained trigger activity or torsade de pointes-like events were observed only in ARVC-hiPSC-CMs (Figure 5). All NDPK-B effects were abolished in the additional presence of PHP-1 (Figure 4D,E).

### 3.4. A Specific SK4 Channel Blocker Suppressed Cell Beating

**Figure 6:**
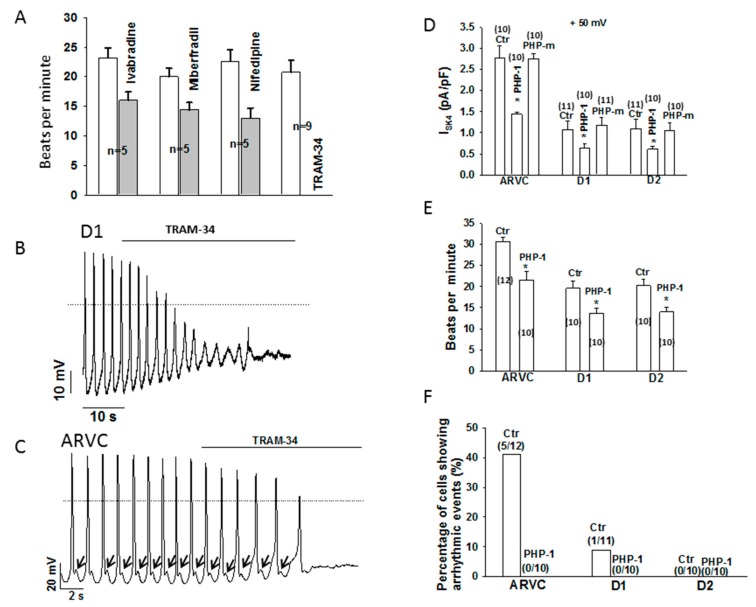
The SK4 channel blocker TRAM-34 and PHP-1 suppressed the cell beating and the occurrence rate of arrhythmic events. A channel specific blocker was applied to cells through a perfusion pipette. The recombinant PHP-1 (100 ng/ml) or the enzyme inactive mutant form of PHP-1 (PHP-m, 100 ng/mL) was applied into cells through the patch pipette. The spontaneous action potentials (sAPs) and I_SK4_ were recorded before and after the application of the drug. (**A**) Mean values of beating frequency of healthy donor (D1) cells in the absence and presence of 3 µM ivabradine (blocker of the funny channel), 10 µM mibefradil (T-type calcium channel blocker), 10 µM nifedipine (L-type calcium channel blocker) or 1 µM TRAM-34. (**B**) An example of sAPs in a D1-hiPSC-CM showing that TRAM-34 suppressed cell beating. (**C**) An example of sAPs in an ARVC-hiPSC-CM showing that TRAM-34 suppressed both the cell beating and the delayed afterdepolarizations (black arrows). (**D**) Mean values of I_SK4_ in donor and ARVC-cells showing that PHP-1 but not its inactive mutant decreased I_SK4_. (**E**) Mean values of beating frequency in donor and ARVC-cells showing that PHP-1 reduced the frequency (pacemaker activity) of cells. (**F**) Mean values of the occurrence rate of arrhythmic events in donor and ARVC-cells showing that PHP-1 prevented arrhythmic events. The numbers given represent cell numbers, * *p* < 0.05.

It has been shown that SK4 channels may contribute to pacemaker activity [9]. Therefore, the effects of SK4 blockade on spontaneous action potentials (APs) were assessed to examine possible functions of SK4 channels for the automaticity of hiPSC-CMs. While the SK4 ion channel blocker TRAM-34 (1 µM) either strongly suppressed or completely terminated the spontaneous APs in both donor and ARVC cells (Figure 6A–C), the I_f_ blocker ivabradine (3 µM), T-type Ca^2+^ channel blocker mibefradil (10 µM) and L-type Ca^2+^ channel blocker nifedipine (10 µM) reduced the frequency of APs only slightly (Figure 6A). These data indicate a major contribution of SK4 channels to the pacemaker activity of hiPSC-CMs. Furthermore, TRAM-34 also suppressed the arrhythmic events, including delayed afterdepolarizations (Figure 6C).

### 3.5. PHP-1 Reduced I_SK4_, Pacemaker Activity and the Occurrence of Arrhythmic Events in hiPSC-CMs

To test for a possible pathogenic role of endogenous NDPK-B in the enhanced I_SK4_, pacemaker activity and arrhythmic events observed in ARVC-hiPSC-CMs, PHP-1 alone was applied into cells and the aforementioned measurements were repeated. The application of PHP-1 (100 ng/mL) but not the enzyme-inactive mutant PHP-1 H53A (100 ng/mL) decreased I_SK4_ significantly (Figure 6D). In addition, the beating frequency was reduced and arrhythmic events were prevented by PHP-1 (Figure 6E,F). These data point to an important role of protein histidine phosphorylation induced by endogenous NDPK-B for pacemaker activity and arrhythmogenesis in ARVC.

## 4. Discussion

In this study, we investigated the role of NDPK-B and SK4 channels for arrhythmogenesis in ARVC using hiPSC-CMs from a patient with ARVC carrying a missense mutation (p.Gly638Arg) in the DSG2. We demonstrate for the first time that (i) both NDPK-B and SK4 expressions are elevated in ARVC cardiomyocytes; (ii) the application of recombinant NDPK-B into hiPSC-CMs activated SK4 channels enhances both pacemaker activity and arrhythmic events; and (iii) PHP-1 as a phosphohistidine-specific phosphatase acting on SK4 channels suppresses the enhanced pacemaker activity and prevents the occurrence of arrhythmic events in ARVC-hiPSC-CMs.

The frequently discussed mechanism underlying tachyarrhythmias in ARVC is the conduction slowing between cells caused by cell-detaching or intercellular fibrosis plus fat-deposit. However, some studies demonstrated that electrophysiological changes precede structural changes [18], suggesting that other possible arrhythmogenic mechanisms are involved in ARVC. We observed reduced sodium channel currents and abnormal APs with reduced Vmaxs in cells from the ARVC patient [6]. Vmax is important for the conduction of the excitation in and between cells. Therefore, reduced Vmax can cause a slowing down of the conduction and may lead to tachyarrhythmias in patients with ARVC. However, mechanisms other than the conduction defect might contribute to the phenotype.

In the current study, we detected enhanced expression and currents of SK4 channels in ARVC-hiPSC-CMs, which have been shown before to contribute to pacemaker activity in embryonic stem cell derived cardiomyocytes and hiPSC-CMs as well as mouse cardiomyocytes [8,9]. In addition, we also observed higher automaticity and occurrence rate of arrhythmic events, which obviously involve I_SK4_ in ARVC-hiPSC-CMs. The contribution of SK4 channels to pacemaker activity and arrhythmogenesis was also observed in CPVT-hiPSC-CMs [8]. Considering that SK4 channel activity is related to the occurrence of arrhythmic events in both CPVT- and ARVC-hiPSC-CMs and important for cell automaticity, we speculate that the elevated pacemaker activity in cardiomyocytes can contribute to arrhythmogenesis in patients with ARVC. It is well-known that SK4 channels exist in embryonic stem-derived cardiomyocytes, iPSC-CMs and mouse pacemaker cells [8]. Whether SK4 channels are expressed in human ventricular cardiomyocytes, especially in “diseased” cardiomyocytes in patients with ARVC, is not known. If the SK4 channel expression in diseased cardiomyocytes is upregulated together with NDPK-B, as shown here for ARVC-hiPSC-CMs, the automaticity of the cells will be enhanced and the likelihood of ectopic beats will be increased. Therefore, both the enhanced ectopic excitations and conduction defect caused by structural abnormalities or ion channel dysfunctions might contribute to the occurrence of tachyarrhythmias in ARVC.

How SK4 channels influence pacemaker activity is still not fully explored. It has been hypothesized that activation of SK4 channels leads to an enhanced K^+^ efflux resulting in hyperpolarization of cell membrane. The hyperpolarization increases the driving force for influx of positively charged ions conducted by hyperpolarization activated channels (HCN, also called I_f_ channels) or T-type or L-type calcium channels. The enhanced inward current accelerates the diastolic depolarization and enhances automaticity (pacemaker activity). In hiPSC-CMs, however, I_f_ channel expression is low and I_f_ current is small [19]. More strikingly, an I_f_ blocker (ivarbradine), a T-type calcium channel blocker (mibefradil) and an L-type calcium channel blocker (nifedipine) all failed to largely reduce the automaticity, suggesting that those ion channels are, alone, not critical for the pacemaker activity in hiPSC-CMs. Another study showed that HERG channel currents determined the MDP (maximum diastolic potential) but not the pacemaker activity in hiPSC-CMs [20]. Nevertheless, the current study demonstrates that SK4 currents are important for the pacemaker activity in hiPSC-CMs as the SK4 ion channel blocker TRAM-34 largely or completely inhibited the automaticity of both donor and ARVC-hiPSC-CMs.

The next question needing to be addressed is how the SK4 channel expression and current are enhanced in ARVC-cells. Our recent study showed that the intracellular Ca^2+^-concentration in ARVC-hiPSC-CMs was the same as that in donor cells [6], suggesting that the increased SK4 currents resulted from a regulation other than an elevated intracellular Ca^2+^ concentration required for the calmodulin-dependent regulation of SK4. Besides Ca^2+^, several protein kinases, including PKA, PKG, PKC and NDPK-B, have been shown to regulate SK4 channels. Among them, NDPK-B seems to be the most important regulator for SK4 channel activity. The activation of SK4 channels by NDPK-B has been well established in different cell systems, including HEK cells, lymphocytes and smooth muscle cells [10,11,12,17]. It is known that NDPK-B can phosphorylate the histidine residue at the position 358 (His358) in the SK4 channel [12]. In the presence of Ca^2+^, upon phosphorylation of His358, copper ion binding in the channel is abrogated, and the calcium-induced conformational changes in the calmodulin-binding domain lead to channel opening [10]. The histidine phosphatase PHP-1 can dephosphorylate His358 and counteract the effects of NDPK-B in all cell types analyzed so far. In our ARVC-hiPSC-CMs, NDPK-B expression at both mRNA and protein levels was upregulated, suggesting the possibility that the increased I_SK4_ in ARVC-hiPSC-CMs may result from the enhanced histidine phosphorylation in SK4 channels by NDPK-B, aside from an increased channel density. Indeed, recombinant NDPK-B but bot NDPK-A or NDPK-C, applied into hiPSC-CMs through the patch pipette, enhanced I_SK4_, and additional application of PHP-1 abolished the effect of NDPK-B. Importantly, application of PHP-1 alone also reduced I_SK4_ in hiPSC-CMs, indicating a contribution of endogenous histidine phosphorylation by NDPK-B to I_SK4_. These data demonstrate that SK4 channels in hiPSC-CMs can be activated by NDPK-B. Considering that NDPK-B is an upstream regulator of SK4 channels, and both the SK4 blocker TRAM-34 and NDPK-B counter-actor PHP-1 reduced pacemaker activity and occurrence of arrhythmias in ARVC cells, we speculate that NDPK-B/SK4 upregulation might be a reason for arrhythmogenesis in ARVC. However it remains to be clarified (i) how NDPK-B expression was upregulated based on the desmosomal gene mutation occurring in ARVC, (ii) whether the observed changes in NDPK-B and SK4 are general in all ARVC patients with different gene mutations and (iii) why application of NDPK-B into a cell caused different forms of arrhythmias. Additionally, the possibility that the elevated expression level of NDPK-B may enhance the pacemaker activity and arrhythmias through non-SK4 involving signaling events cannot be excluded. Nevertheless, our data indicate that the SK4-activating effect of NDPK-B can contribute to ectopic activity and the occurrence of arrhythmias in ARVC, at least with the DSG2 mutation (p.Gly638Arg).

In summary, this study revealed that in hiPSC-CMs from an ARVC patient, (i) NDPK-B and SK4 channels were upregulated, (ii) SK4 channels were activated, (iii) pacemaker activity was enhanced and (iv) the occurrence of arrhythmias was increased. We therefore speculate that upregulation of NDPK-B and SK4 can contribute to arrhythmogenesis in ARVC through enhanced histidine phosphorylation of SK4. Thus, NDPK-B may possibly be a potential therapeutic target for treating arrhythmias in ARVC-patients.

## 5. Study Limitations

Some limitations should be considered in extrapolating the data from the current study. The hiPSC-CMs from only two healthy donors and one ARVC-patient were used for this study. Therefore, we cannot rule out the differences among individuals. The results from the patient of this study should, from a statistical point of view, not be interpreted as that from the whole population of ARVC-patients. From a practical point of view, it is difficult to include several patients with the same mutation in the same gene, and hence, for the present study it was not feasible to include cells from second and third ARVC patients to increase the power. Patients with ARVC carrying other DSG2 mutations or mutations in other genes were not recruited for this study, and therefore, whether NDPK-B and SK4 upregulation exist in other ARVC-patients with different gene mutations as well needs to be clarified in further studies. In addition, immaturity is a well-known limitation of hiPSC-CMs. The differences of cell properties, including electrical activities between hiPSC-CMs and native cardiomyocytes, should be also considered in interpreting the data of this study. Furthermore, due to the limitation of availability, the native cardiac cells from ARVC-patients were not investigated for this study. Whether the upregulation of NDPK-B and SK4 is the phenotypic characteristic\in ARVC-patients, which is the most important question for this study, cannot be addressed, although it may be the case, at least in some ARVC-patients with a certain gene mutation. Nevertheless, this study may trigger further studies in this area.

## Supplementary Materials

The following are available online at https://www.mdpi.com/2077-0383/9/2/486/s1, Figure S1: Expression of cardiac markers in ARVC-hiPSC-CMs. Figure S2: Assessment of the enzymatic activities of recombinantly expressed NDPK isoforms compared to catalytically inactive point mutants.
