# Supplementary material for: Nucleoside Diphosphate Kinase B Contributes to Arrhythmogenesis in Human-Induced Pluripotent Stem Cell-Derived Cardiomyocytes from a Patient with Arrhythmogenic Right Ventricular Cardiomyopathy
Source: J Clin Med. 2020 Feb 10;9(2):486. doi: 10.3390/jcm9020486 (PMC7073527; doi:10.3390/jcm9020486)
Supplement: Supplementary file 1 [file jcm-09-00486-s001.pdf]

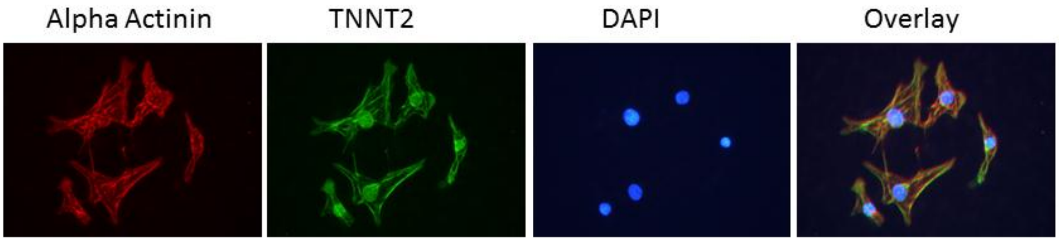

Supplementary Figure S1: Expression of cardiac markers in ARVC-hiPSC-CMs. Shown are representative images of immunohistochemical proof showing successful differentiation of cardiomyocytes by the expression of cardiac markers alpha-actinin (red) and troponin T (TNNT2, green) in ARVC-hiPSC-CMs. Cell nuclei were stained by DAPI.

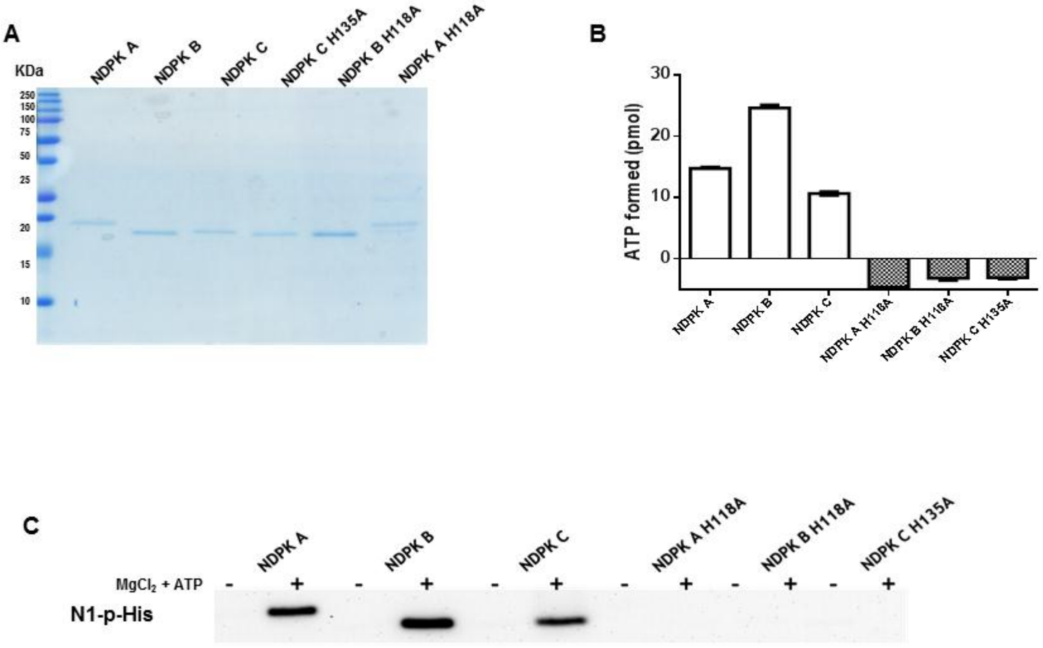

Supplementary Figure S2. Assessment of the enzymatic activities of recombinantly expressed NDPK isoforms compared to catalytically inactive point mutants. A**.** 50 ng of purified His_6- tagged NDPK A, B or C or there inactive mutants were separated by 15% SDS-PAGE and visualized with coomassie brilliant blue R-250. B. The NTP/NDP transphosporylase activity of 90 pM of the indicated recombinant NDPKs was quantified by the ATP formation from 10 µM ADP and 100 µM GTP as substrates. Mean ± SEM from four assay replicates are shown. C. Representative western blot showing the autophosphorylation of NDPKs on their catalytic histidine residue: 100 ng of NDPKs were incubated with and without 1 mM Mg^{2+}-ATP, separated by 15% SDS PAGE, and transferred onto a nitrocellulose membrane. The blot was probed with a N1-phospo-histidine antibody specifically recognizing the histidine residues phosphorylated at the N1 position.
